# The influence of distal and proximal muscle activation on neural crosstalk

**DOI:** 10.1371/journal.pone.0275997

**Published:** 2022-10-25

**Authors:** Yiyu Wang, Osmar Pinto Neto, Madison M. Weinrich, Roberto Castro, Traver Wright, Deanna M. Kennedy

**Affiliations:** 1 Department of Kinesiology and Sport Management, Texas A&M University, College Station, Texas, United States of America; 2 Biomedical Engineering Department, Anhembi Morumbi University, São Paulo, State of São Paulo, Brazil; University of Hartford College of Education Nursing and Health Professions, UNITED STATES

## Abstract

Previous research has indicated that neural crosstalk is asymmetric, with the dominant effector exerting a stronger influence on the non-dominant effector than vice versa. Recently, it has been hypothesized that this influence is more substantial for proximal than distal effectors. The current investigation was designed to determine the effects of distal ((First Dorsal Interosseous (FDI)) and proximal (triceps brachii (TBI)) muscle activation on neural crosstalk. Twelve right-limb dominant participants (mean age = 21.9) were required to rhythmically coordinate a 1:2 pattern of isometric force guided by Lissajous displays. Participants performed 10, 30 s trials with both distal and proximal effectors. Coherence between the two effector groups were calculated using EMG-EMG wavelet coherence. The results indicated that participants could effectively coordinate the goal coordination pattern regardless of the effectors used. However, spatiotemporal performance was more accurate when performing the task with distal than proximal effectors. Force distortion, quantified by harmonicity, indicated that more perturbations occurred in the non-dominant effector than in the dominant effector. The results also indicated significantly lower harmonicity for the non-dominant proximal effector compared to the distal effectors. The current results support the notion that neural crosstalk is asymmetric in nature and is greater for proximal than distal effectors. Additionally, the EMG-EMG coherence results indicated significant neural crosstalk was occurring in the Alpha bands (5–13 Hz), with higher values observed in the proximal condition. Significant coherence in the Alpha bands suggest that the influence of neural crosstalk is occurring at a subcortical level.

## Introduction

The ability to coordinate bimanual actions involving distal and proximal effectors is important for many activities associated with daily living. For example, tasks such as tying shoes or buttoning a shirt require coordinated actions between two distal (e.g., fingers, wrists) effectors, whereas tasks such as driving a car or placing a pan in an oven often require a coordinated effort with more proximal effectors (e.g., arms, shoulders). A large body of research has focused on the control of coordinated actions [1–12]. Such work has used a variety of effectors (e.g., fingers, wrists, arms, shoulders) to examine coordination dynamics [3, 12]. However, investigations directly comparing the coordination of distal and proximal effectors are less common [13, 14].

The general results of research investigating the control of bimanual actions has demonstrated only two inherently stable coordination patterns: in-phase (ϕ = 0°) and anti-phase (ϕ = 180°), while other relative phase (e.g., 90°) and frequency (e.g., 1:2) patterns have proved less stable and more difficult to perform without extensive practice [3, 15–17]. The difficulty in performing complex bimanual tasks such as 90° relative phase or 1:2 multifrequency have been attributed to a coalition of constraints that include neural crosstalk [10, 18]. Neural crosstalk occurs when each hemisphere sends commands to the contralateral effector via crossed corticospinal tracts, interhemispheric structures, or spinal interneurons while concurrently sending a mirror image command to the ipsilateral effector via uncrossed corticospinal tracts, interhemispheric structures, or spinal interneurons [10, 19, 20]. This ipsilateral influence may add to or subtract from the activation of the contralateral effector depending on the characteristics of the signal (e.g., excitatory or inhibitory) [10, 21, 22]. When participants are required to produce bimanual coordination patterns with different actions (e.g., amplitude, direction, frequency, force) for each effector, interference from conflicting information or a partial intermingling of the control signals may account for the stability characteristics and difficulty associated with complex relative phase or multifrequency patterns [22–24].

In a series of experiments, researchers demonstrated bimanual interference consistent with neural crosstalk during a 1:2 bimanual force task [4, 5, 11, 25–28]. The task required participants to produce two patterns of isometric force pulses with the dominant (right) effector for every pulse produced by the non-dominant (left) effector. Perturbations in the force and force-velocity time series were observed for the non-dominant effector that were associated with the activation and release of force by the dominant effector. However, similar distortions in the force and force-velocity time series for the dominant effector that could be attributed to force production by the non-dominant effector were not observed [4]. The results of this line of inquiry are consistent with previous research indicating neural crosstalk is asymmetric, with the dominant effector/hemisphere exerting a stronger influence on the non-dominant effector/hemisphere than vice versa [21, 23, 29, 30].

Research has pointed to issues related to hand dominance as the source of the asymmetry associated with bimanual interference [29]. For example, performance differences between the limbs can be observed in both unimanual and bimanual tasks. Individuals are more accurate and consistent during unimanual tapping tasks with the dominant limb than the non-dominant [30], individuals are more accurate at producing the spatial and temporal goals of a bimanual task with the dominant limb than with the non-dominant limb [16, 31–33], and trajectory distortions and direction reversals most often occur in the non-dominant limb [33, 34]. To further examine whether the influence of force produced by one effector on the contralateral effector during a bimanual multifrequency task was the result of the effector assigned the faster frequency or a bias associated with limb dominance an experiment was conducted comparing performance on a 1:2 and 2:1 bimanual force tasks [5]. Note, a 2:1 bimanual force tasks requires participants to produce two patterns of force with the non-dominant limb for each pattern of force produced with the dominant limb. The results indicated that when the dominant effector performed the faster frequency (1:2 task), distortions in the force and force-velocity time series were observed for the non-dominant effector that could be attributed to the activation of force in the contralateral effector, However, when the non-dominant effector performed the faster frequency (2:1 task), distortions in the force and force-velocity profiles for both effectors were observed. Distortions in the non-dominant effector, when assigned the faster frequency indicated that the source of interference is not limited to limb assignment (effector producing the faster frequency) but also a function of limb dominance. Results were less clear when left limb dominant participants performed the multifrequency tasks [35]. It has been suggested that left limb dominant individuals do not share the same coordination biases as right limb dominant individuals [36]. To reduce coordination biases associated with limb dominance, the current investigation was limited to right limb dominant participants and used a task that required the dominant limb to perform the faster frequency.

Recently, it has been hypothesized that there is a more pronounced bilateral interference in homologous proximal effectors compared to homologous distal effectors [13, 14]. The hypothesis was based upon neuroanatomical evidence indicating cortical and subcortical areas of the brain are distinctly connected with distal and proximal effectors of the body [37–42]. In addition, the number of commissural fibers connecting muscles through the corpus callosum (CC) and in the spinal cord is higher for proximal effectors than for distal effectors [37, 38, 43–45]. Note, research has pointed to interhemispheric interactions through the CC as a possible source of interference observed in complex bimanual tasks [10, 20, 22, 46]. The CC is the primary structure for exchanging information between the hemispheres, which is necessary to coordinate actions between two effectors successfully. Impaired CC function is often associated with a decrease in bimanual coordination [47, 48]. Subcortical structures have also been found to contribute to the integration or cancellation of bimanual control signals. For example, an fMRI study has indicated subcortical areas such as the putamen and the anterior cerebellum were consistently activating when performing bimanual actions [49], and electromyography (EMG) evidence has indicated increased bilateral coherence between the homologous muscles in the alpha bands (5–13 Hz) [11, 50]. Moreover, uncrossed ventromedial corticospinal tracts are associated with the control of proximal effectors, while the crossed lateral corticospinal tracts are associated with the control of distal effectors [39–42]. Therefore, it is possible that bimanual actions involving proximal effectors are exposed to greater interference than distal effectors [13, 14].

Recent experiments comparing bimanual interference in proximal and distal effectors used a task that required participants to trace a sinewave pattern with their dominant proximal (shoulder and elbow) or distal (wrist and fingers) effector with a joystick while the non-dominant effector was inactive (unimanual control), produced a secondary movement task with distal effector, or produced secondary movement task with proximal effector [13, 14]. Yet, the studies only assessed the performance of the dominant limb. It should be noted that bimanual interference typically results in greater perturbations, trajectory distortions, and/or direction reversals in the non-dominant limb [4, 21, 26, 33, 34]. Additionally, when you compare results between experiments using the same task with various effectors, different and sometimes conflicting results are reported. For example, in an experiment using distal effectors to learn a new coordination pattern, changes in intrinsic dynamics were reported [12]. However, no such changes were observed in a follow-up experiment using proximal effectors [3]. The lack of change suggests differences in how the brain controls and learns coordination tasks using different effectors. Thus, many questions related to distal and proximal effector influence on bimanual interference remain unanswered. Therefore, the purpose of the current investigation was to determine the effects of distal ((First Dorsal Interosseous (FDI)) and proximal (triceps brachii (TBI)) muscle activation on bimanual interference using a multifrequency force task.

It has been suggested that force may modulate the strength of neural crosstalk with more bimanual interference associated with higher levels of force production [28, 51]. Thus, coordination tasks that require force production instead of movement may magnify bimanual interference and provide further details regarding constraints that impact bimanual coordination involving distal and proximal effectors. In addition, muscle activity of the involved muscles was analyzed using electromyography (EMG). More specifically EMG-EMG wavelet coherence was used to determine the influence of neural crosstalk on coordination stability and to identify the source (cortical, subcortical) of neural crosstalk [11]. EMG-EMG wavelet coherence is based on the cross-correlation between two EMG signals in the frequency domain and is measured on a scale from 0 to 1. It provides a method to identify periods of common neural input to two effectors [52, 53]. In addition, it can be used to identify the source of neural crosstalk by examining what is happening in specific frequency bands during periods of interference. Significant coherence in the Alpha band (5–13 Hz) indicate subcortical influences, while coherence in Beta (13–30 Hz) and Gamma (30–60 Hz) bands indicate cortical origins [54, 55]. This technique may provide important insights regarding differences between the activation of distal and proximal effectors during the performance of bimanual actions.

Understanding differences between the neural control of distal and proximal effectors and the effects of neural crosstalk is essential for both practical and theoretical purposes. For example, recovery of both hand (distal) and arm (proximal) function in stroke patients is a top priority in clinical practice and research [56]. Rehabilitation protocols utilizing bimanual actions as a therapeutic modality for stroke rehabilitation are common [57–59]. Such protocols are based upon principles of neural crosstalk, also called cross-education or cross-facilitation [19, 60], and use techniques such as mirror therapy [61], robotic assistive devices [62], and transcranial magnetic stimulation (TMS) [63]. Interestingly, differences in such treatment approaches have been observed between distal and proximal muscles [62, 64, 65]. As such, further research is warranted to fully understand the differences in proximal and distal muscle activation on neural crosstalk.

## Methods

### Participants

Twelve young adults (Mean age = ±21.9, SD = ± 3.03; Male = 4, Female = 8) volunteered to participate in the experiment. Based upon previous research only right limb dominant individuals were recruited. The Edinburgh Handedness Inventory was used to confirm all participants were right limb dominant [66]. The Institutional Review Board at Texas A&M University approved the procedures, and participants provided written informed consent before participation in the study.

### Apparatus

**Fig 1:**
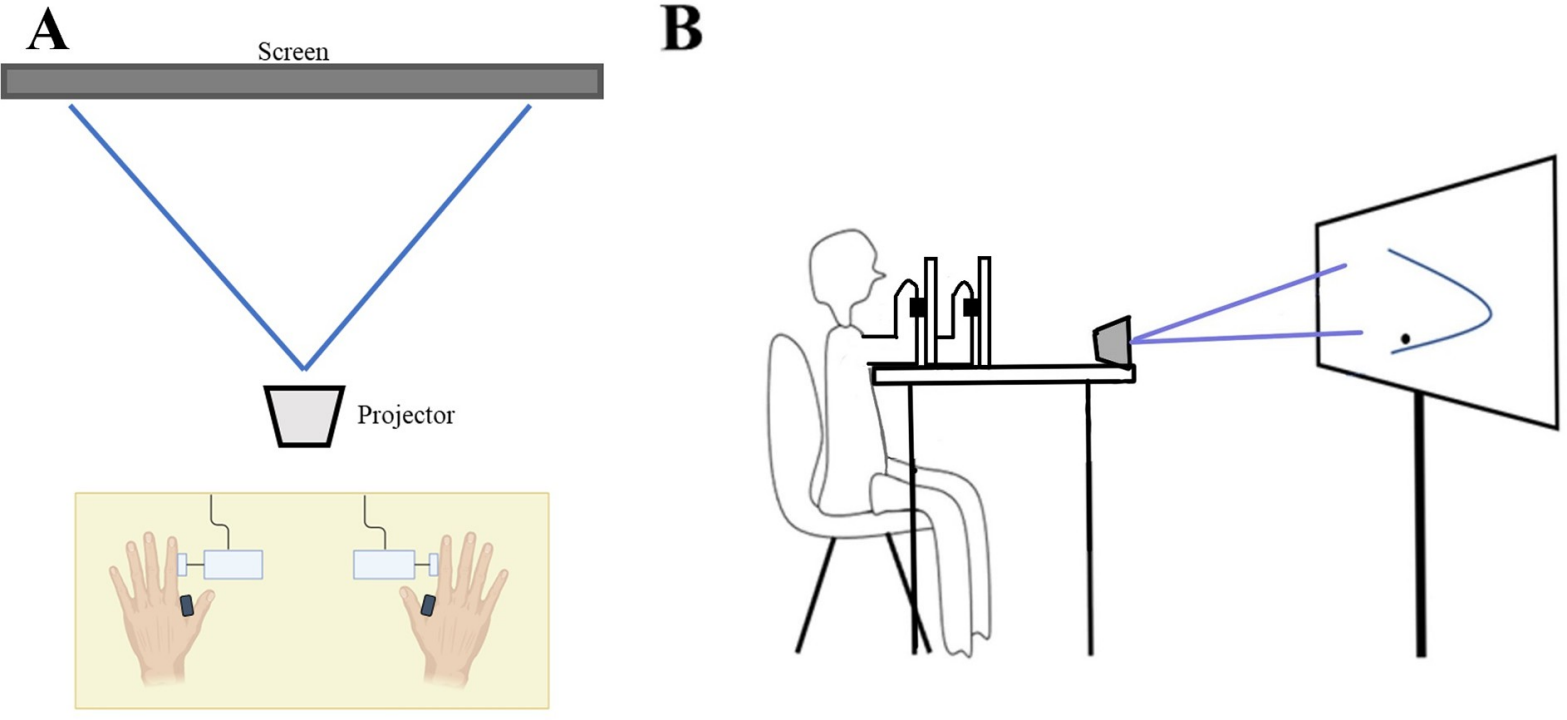
Experimental setup. (a) Illustration depicting experimental setup for bimanual task utilizing distal (FDI), and (b) proximal (TBI) muscles.

Separate experimental setups were designed to test coordination of force production between non-dominant and dominant limbs using both distal (FDI) and proximal (TBI) muscles. For the distal setup, a custom mirror-image apparatus was designed for each limb to isolate FDI contractile force of the index finger towards the thumb (Fig 1). The seat and test table height were adjusted so that the subjects’ hands rested comfortably on the table in front of them approximately shoulder width apart. Each hand was placed flat on a testing table with the medial side of each index finger placed against a force transducer. The thumb was hooked around a barrier with an adjustable span for varied hand size to open the gap between the index finger and thumb. A separate barrier between the index and middle fingers isolated FDI contractile force against the transducer and prevented subjects from using their whole hand or wrist to modulate force. Velcro straps were used to secure the index finger to the apparatus.

For the proximal setup, a custom mirror-image apparatus was designed for each limb to isolate TBI contractile force used to extend the lower arm at the elbow (Fig 1). The height of the chair and table were adjusted so that both elbows could rest comfortably on the testing table slightly below shoulder level (Fig 1). The elbows rested in a padded cup that provided a positive stop, fixing the elbow in position. A force transducer mounted directly above the elbow cup was adjusted to the height of the wrist. The positive stop of the padded elbow cup and static force transducer location isolated force production to elbow extension via TBI contraction.

The forces exerted against the transducers were converted into a voltage representing the instantaneous value of the applied force. The voltages were recorded using an AD converter (NI USB-6210 Board, National Instruments Corp, Austin, TX, USA) installed on the computer programmed to sample at 200 Hz. A force template and a cursor representing the applied forces were displayed on a 50-inch canvas using a projector directly in front of the participants. A wireless electromyography (EMG) system (Delsys Inc., Boston, MA, USA) was utilized to record the neural input from the triceps brachii muscles (TBI) and the first dorsal interosseous (FDI) muscles. The EMG signals were sampled at 2000 Hz and synchronized with the real-time force values.

### Procedure

Before entering the testing room, participants were assigned to start in either the distal or proximal condition. In the distal condition, participants sat at a table with their forearms resting on a testing table. The arms were positioned shoulder-width apart while the fingers were resting against force transducers. Participants were asked to abduct their non-dominant and dominant index fingers to push against force transducers mounted on the right and left sides of the testing table. In the proximal condition, participants were asked to place their forearms against corresponding force transducers. The force transducers were adjusted so that the participant’s wrist contacted the load cell to produce isometric force using the non-dominant and dominant proximal effectors. The elbow was positioned at a 90° angle while the wrists were in a neutral position. Participants were required to maintain the correct position throughout each trial.

Participants’ maximum voluntary contraction (MVC) with both distal and proximal effectors were measured. Participants followed verbal instructions to activate their distal and proximal effectors from baseline to maximum over a 2-s period and to maintain the maximal force for approximately 3 s. The MVC force was calculated as the mean force based on three MVC trials and rounded to the lowest integer. The maximum force required for the coordination task was set at 20% MVC.

**Fig 2:**
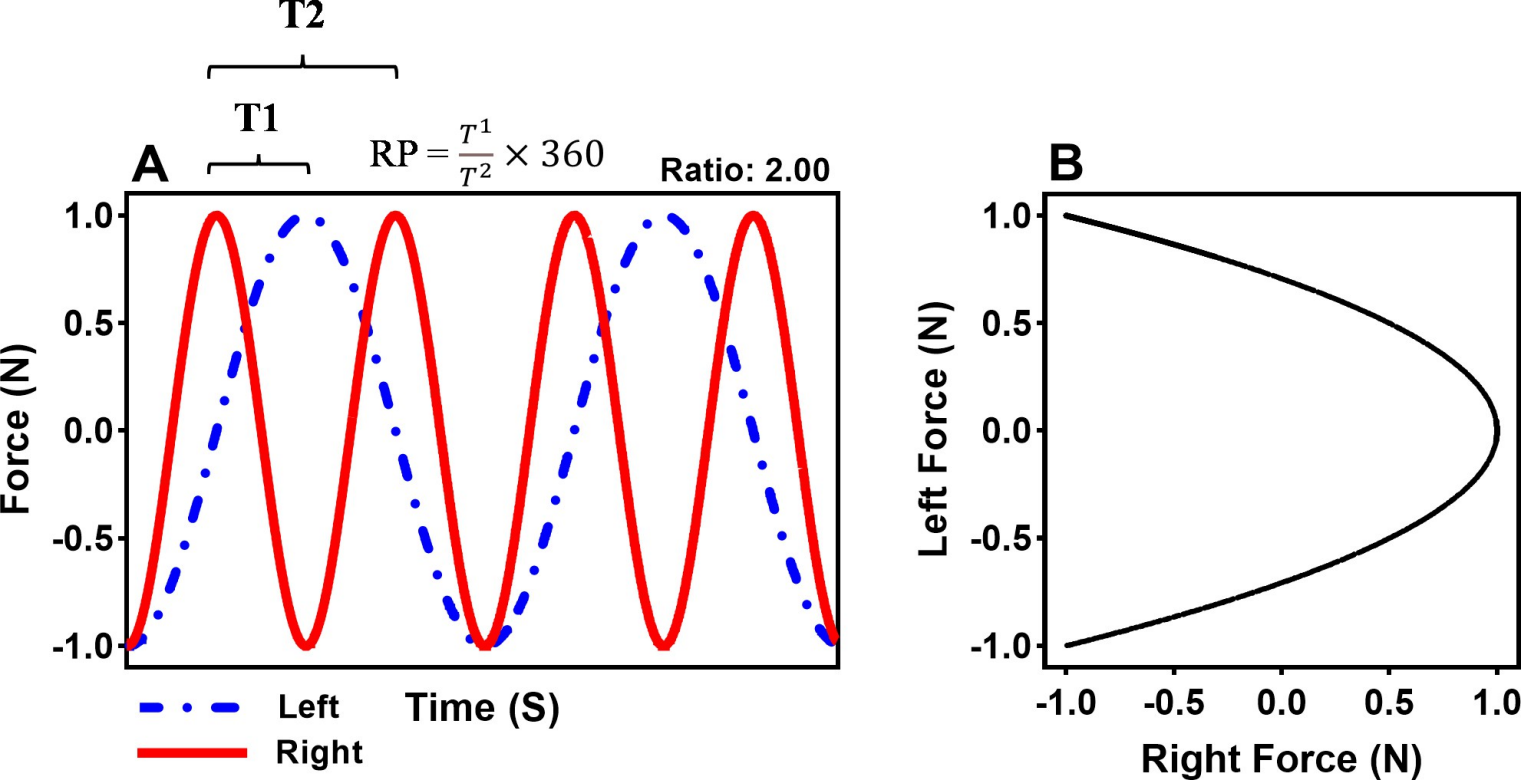
Task description. (a) Simulated goal coordination pattern for non-dominant and dominant limb during 1:2 coordination task, with (b) corresponding Lissajous template for 1:2 task. Note, the calculation for relative phase is also included.

Participants were required to rhythmically produce a pattern of isometric force on the left side transducer with the non-dominant effector that was coordinated with the pattern of isometric forces produced on the right-side force transducer with the dominant effector in a 1:2 force coordination pattern using Lissajous display information to guide performance. Lissajous display information consisted of a Lissajous plot that incorporated a goal template and a cursor indicating the forces produced by both effectors. The Lissajous display was generated by plotting two Sine functions in a 2-dimensional plane. The cursor moved from left to right as force was produced with the dominant effector and from bottom to top as force was produced by the non-dominant effector. The goal template illustrated the specific pattern of required forces to produce the goal pattern (Fig 2). Participants were instructed to follow the general shape of the template with the cursor. Following the template with the cursor resulted in the production of two patterns of force with the dominant effector for every pattern of force produced by the non-dominant effector (Fig 2). After any trial in which the average frequency of the dominant effector was below 1.0 Hz, the experimenter encouraged the participants to increase the speed with which they produced the patterns of force without disrupting the goal pattern. Participants performed ten practice trials for each condition (distal and proximal) in a counterbalanced order. Upon completing all practice trials, participants were given four test trials to assess bimanual performance of the 1:2 pattern. Surface EMG was placed on the muscle belly of the acting effectors to record neural input during the testing trials. The order of testing trials was also counterbalanced. All trials were 20 s with a 10 s rest period between trials, and each condition was separated by a 2 min rest period.

### Measures and data reduction

All data reduction was performed using MATLAB (R2020a). A low-pass filter with a second-order dual-pass Butterworth was applied to clean the noise in the force signal. The cutoff frequency for the low-pass filter was set as 10 Hz. A 3-point difference algorithm was used to compute force-velocity and acceleration signals from the limb’s force time series. Then, the signals were normalized to a range from -1 to 1 by their maximum (positive or negative) values for each half cycle.

#### Unimanual force measures

Force harmonicity is used to quantify the distortion in force time series [4, 11, 26, 67]. Harmonicity quantifies the harmonic nature of force produced by each effector for every half cycle based upon zero-crossings. An index of harmonicity is computed using non-overlapping windows between pairs of force-velocity zero-crossings [68]. When the force acceleration trace of each half-cycle only contained a single peak, the value of harmonicity was set to 1. When an inflection occurred in the half-cycle force acceleration trace, harmonicity was computed as the minimum ratio to maximum acceleration. When the half-cycle acceleration trace switched direction (crossed from positive to negative or vice versa) within this window, the harmonicity value was set to 0. Next, the harmonicity value for a trial was averaged from the harmonicity from each half cycle. A harmonicity index of 1 indicates a harmonic force production time series where bimanual interference is less likely to occur. In contrast, a harmonicity index of 0 indicates that bimanual interference has likely impacted the bimanual force production for the effector.

Inter-peak interval, inter-peak interval variability, and phase angle velocity were also calculated. Inter-peak interval was computed as the mean of all inter-peak intervals within a trial. Inter-peak interval variability was defined as the standard deviation of the inter-peak intervals within a trial. The inter-peak intervals and inter-peak intervals variability indicated the temporal accuracy and the temporal stability between two consecutive force peaks (inter-peak interval = forcepeakn+1 –forcepeak_n_). Phase angle velocity was computed to determine the speed of force pulse per second. The phase angle (θ_n_) for each effector (n = r, l) was computed for each sample of the normalized force time series as follows (Eq 1):

$$\theta_{n}={tan}^{-1}\left(\frac{\frac{dX_{n}}{dt}}{X_{n}}\right)$$ (1)

where X_n_ is the normalized force of the non-dominant and dominant effector and dX_n_/dt (d represents derivative) is the instantaneous normalized force velocities for the dominant and non-dominant effector. Then, the phase angles θ_n_ for individuals were unwrapped by finding multiples of 2π and adding appropriate multiples of 2π to each data point following the jump. Phase angles were determined for both the non-dominant and dominant effectors. A 3-point difference algorithm computed phase angle velocity. The phase angle velocity of a dominant effector in 1:2 condition should be roughly two-fold of the non-dominant effector.

#### Bimanual measures

Two independent temporal measures of bimanual coordination were calculated, one based on point estimates of mean cycle duration (inter-peak interval ratio) and the other based on the effector’s continuous phase angles (phase angle slope). Inter-peak intervals for the dominant and non-dominant effectors were used to determine point estimates of mean cycle duration and compute a ratio of the dominant effector cycle duration to the non-dominant effector cycle duration. This measure provides a temporal measure of goal attainment independent of effector coordination tendencies and actual effector force trajectories. An inter-peak ratio of 2.0 would indicate that the interval for the dominant effector was twice that of the non-dominant effector.

Regression analyses of the continuous relative phase angles (see calculation methods above) for the dominant and non-dominant effectors were calculated to determine the slope of the unwrapped non-dominant and dominant effector phase angles across the trial for each participant. This measure is intended to examine the continuous spatial-temporal coordination of the effector forces. The average slope and R^2^ were then determined. The slope and R^2^ of the unwrapped dominant effector phase angle to non-dominant effector phase angles provide a continuous measure of bimanual goal attainment. As with the inter-peak interval ratio, the goal phase angle slope ratio for the 1:2 is 2.0, with no variability.

To determine the accuracy of the task performance, absolute error (AE) was calculated by comparing the relative phase angle (RP) to the goal phase angle. The point-estimate approach (Fig 2) was utilized to compute the RP for the 1:2 tasks. A 3-point difference algorithm was used to identify the peak within each cycle for the non-dominant and the dominant limb. Then the RP was calculated as follows (Eq 2):

$$\frac{tR(n+1)-tL(n)}{tR(n+1)-tR(n)}\times 360$$ (2)

**Fig 3:**
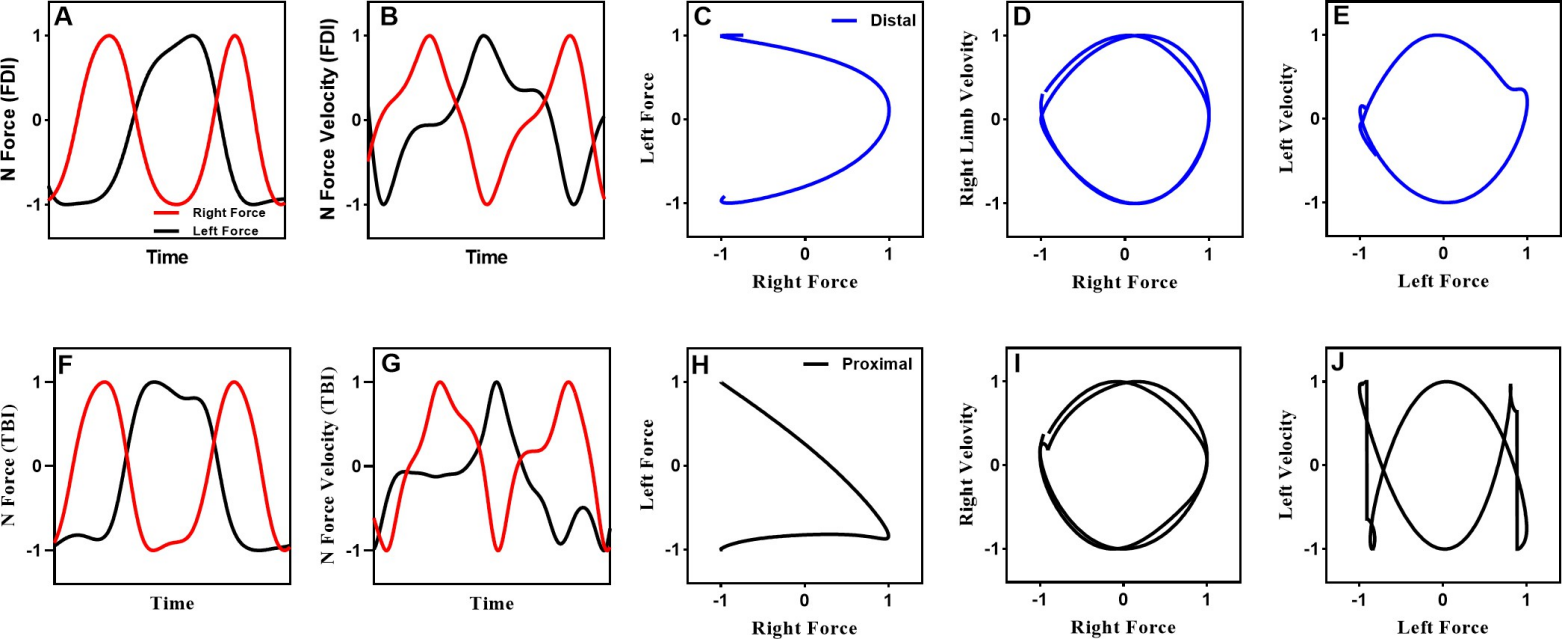
Example performance. (a—e) Example of one cycle of the 1:2 multifrequency task performed with distal effectors and (f—j) one cycle of the task performed with proximal effectors. The figure includes (a, f) non-dominant and dominant effector forces time series, (b, g) non-dominant and dominant effector force velocity time series, (c, h) force-force plots, (d, i) force-force velocity for dominant effector, and (e, j) force-force velocity for non-dominant effector.

Where tR represents the instantaneous time for the dominant limb, and tL means the instantaneous time for the non-dominant limb. The peaks used in the calculation served as critical points to assess the accuracy of the temporal relationship between two active effectors. For the 1:2 task, the RP between the two effectors at the peaks should be 180° if the temporal relationship of force production was accurate. Then RP were averaged for a trial, and AE was calculated by taking the absolute value of the subtraction between the RP and the goal phase angle (180° for the 1:2). A smaller number indicated more accurate movements between two effectors (Fig 2). In addition, force time series, force velocity time series, force-force, and force-force velocity were plotted to provide an example for a comparison between distal and proximal effector’s bimanual performance (Fig 3).

#### EMG measures

All surface EMG signals were band-passed and filtered at 5–500 Hz (4th-order Butterworth). After the high pass filtering process, EMG-EMG wavelet coherence was determined using high-pass filtered at 250 Hz rectified (HPRECT) signals [50, 52, 69]. *Morlet* wavelet transform was utilized to calculate the EMG wavelet frequency domain [70, 71]. Wavelet and cross-wavelet spectra were plotted using Matlab functions developed by Torrence and Compo [72]. The wavelet coherence spectrum [73] was quantified as localized correlation coefficients in the wavelet time-frequency space as follows (Eq 3):

$$WCS(s,\tau {)}^{XY}=\frac{{\left|S(s^{-1}W^{XY}(s,\tau ))\right|}^{2}}{S(s^{-1}{\left|W^{X}(s,\tau )\right|}^{2}).S(s^{-1}{\left|W^{Y}(s,\tau )\right|}^{2})}$$ (3)

where *s* represents the dilation parameter (scale shifting), τ represents the location parameter (time shifting), W^xy^ represents the complex cross wavelet transform of signals X and Y, and S is naturally designed as a smoothing operator with a similar footprint as the *Morlet* wavelet [74]. The wavelet coherence spectrum shows the correlation between the common frequencies of two signals with values ranging from 0–1. Wavelet peak coherence was quantified for three frequency bands: 5–13 (Alpha band), 13–30 (Beta band), 30–60 Hz (Gamma band) [54, 55].

### Statistics

Unimanual measures (harmonicity, inter-peak interval, inter-peak interval variability, and phase angle velocity) for each effector were analyzed in an Effector (distal, proximal) × Limb (non-dominant, dominant) two-way ANOVAs repeated measure on all independent variables. A paired t-test was used to compare the difference of the bimanual dependent variables (AE, the mean inter-peak interval ratio, and the mean phase angle slope) between distal and proximal effectors. In addition, EMG-EMG coherence was analyzed in the Effector (distal, proximal) × Frequency bands (5–13, 13–30, 30–60 Hz) two-way ANOVAs repeated measure on Effector and Frequency bands. Bonferroni’s procedure was used for all post hoc analyses. The Pearson correlation was performed to identify the association between EMG-EMG coherence and harmonicity in the non-dominant effectors. Statistics were performed with the PASW Statistics 18.0 package (SPSS Inc, Chicago, Illinois). The alpha level for all statistical tests was 0.05.

## Results

### Unimanual measures

#### Harmonicity

**Fig 4:**
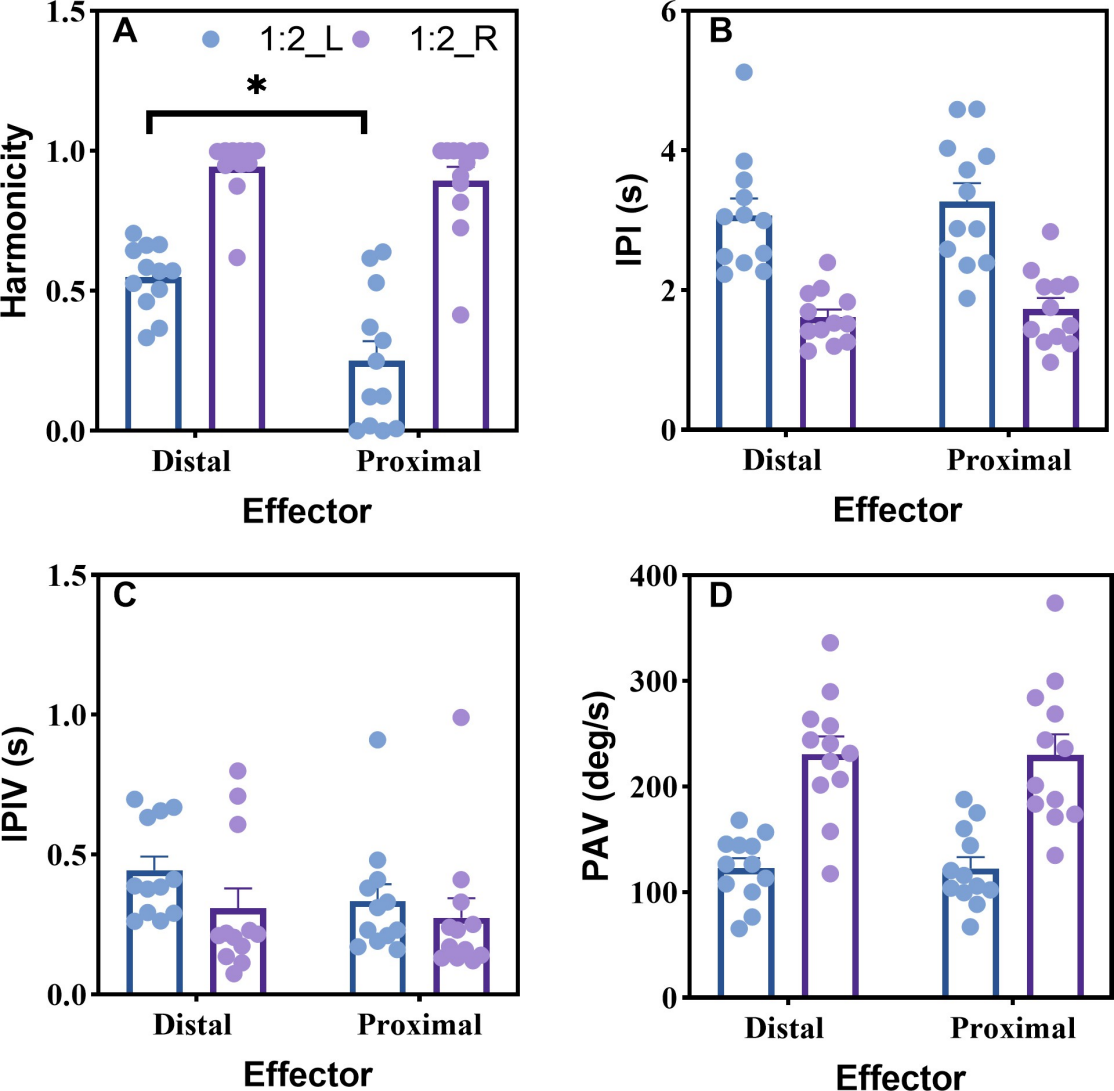
Unimanual measures. (a) Force harmonicity, (b) mean inter-peak interval, (c) standard deviation of inter-peak interval, and (d) phase angle velocity. The error bars represent standard error of the mean (SEM) and * depicts a statistical significance level of less than 0.05. Note that harmonicity for the non-dominant effector was significantly lower for the proximal condition compared to the distal condition.

The analysis indicated a main effect of Effector, F(1, 11) = 17.198, p = 0.002, η2p = 0.610, Limb, F(1, 11) = 111.935, p < 0.001, η2p = 0.911, and Effector × Limb interaction, F(1, 11) = 21.241, p < 0.001, η2p = 0.659. A *post hoc* approach was further used to compare the mean between effectors (distal, proximal) for each limb, and the result indicated a significant difference between distal and proximal effector in the non-dominant limb. Fewer force distortions were observed in the non-dominant limb while using distal effectors (M = 0.550, STD = 0.118) as compared to proximal effectors (M = 0.250, STD = 0.244) (p < 0.001). The lower value of harmonicity (0–1) indicates more adjustments, hesitation, and perturbation occurred during the force production (Fig 4).

#### Inter-peak interval (IPI)

The analysis indicated a main effect of Limb, F(1, 11) = 176.206, p < 0.001, η^2^p = 0.941. The Limb effect, as would be expected for the 1:2 task, indicated longer inter-peak intervals for the non-dominant effector (results, considering both effector groups) compared to the dominant effector (Fig 4).

#### Inter-peak interval variability (IPIV)

The statistical analysis indicated that there was no significant main effect of Effector, F(1, 11) = 2.231, p = 0.163, Limb, F(1, 11) = 2.482, p = 0.143, and Effector × Limb, F(1, 11) = 0.247, p = 0.629. This result indicated that participants can stably produce the goal coordination pattern (Fig 4).

#### Phase angle velocity (PAV)

The analysis indicated a main effect of Limb, F(1, 11) = 125.202, p < 0.001, η2p = 0.919. However, no significant main effect was detected in Effector and Effector × Limb interaction. This indicates that participants performed the task correctly regardless of the effectors used in the task (Fig 4).

### Bimanual measures

**Fig 5:**
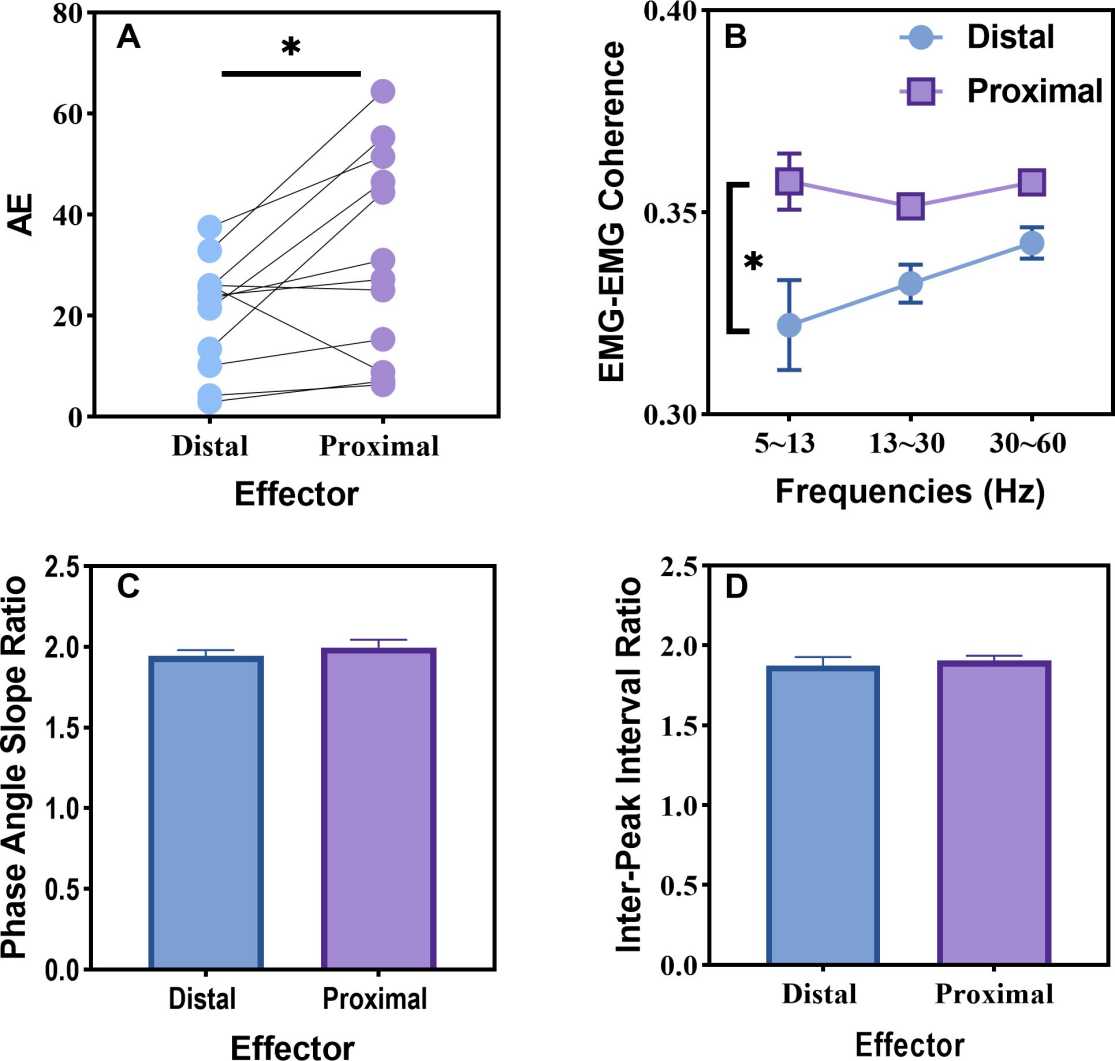
Bimanual measures. (a) Absolute error for distal and proximal effector, (b) EMG-EMG coherence for distal and proximal effector in the alpha (5–13 Hz), beta (13–30 Hz), and gamma (30–60 Hz) frequency bands, (c) phase angle slope ratio, and (d) inter-peak interval ratio. The * indicates a significance level less than 0.05.

**AE.** A paired samples t-test detected a significant difference in AE between distal effectors (M = 20.657, STD = 10.791) and proximal effectors (M = 31.936, STD = 20.225) to produce the 1:2 coordination pattern (t = -2.571, p = 0.026). This result suggested that participants were more accurate when producing the task with distal effectors than proximal effectors (Fig 5).

#### EMG-EMG coherence

The repeated ANOVA analysis indicated a main effect of Effector, F(1, 11) = 10.885, p = 0.007, η^2^_p_ = 0.497. The overall EMG-EMG coherence in all frequency bands tended to be higher when performing the 1:2 coordination pattern with proximal effectors than performing with distal effectors. However, the analysis failed to detect the main effect of Frequency bands and Effector × Frequency bands interaction. A paired sample t-test was conducted to test whether EMG-EMG coherence differed in the alpha bands between distal and proximal effectors. The result indicated a significant difference (p = 0.022) in the alpha band coherence between using distal effector (M = 0.322, STD = 0.039) and using proximal effectors (M = 0.358, STD = 0.024) to produce the bimanual coordination task (Fig 5).

#### The mean phase angle slope (MPAS)

A paired samples t-test also failed to indicate the difference in MPAS between the distal and proximal effector. The slope ratio between the non-dominant effector and the dominant effector was nearby 2.0 for distal effectors (M = 1.946, STD = 0.118) and for proximal effectors (M = 1.996, STD = 0.168). This result further indicated that participants were able to produce the goal coordination pattern regardless of the effector group. (Fig 5).

#### The mean inter-peak interval ratio (MIPIR)

A paired samples t-test failed to detect the difference in MIPIR between distal and proximal effectors. The IPI ratio between the dominant effector and the non-dominant effector was approaching to 2.0 with distal effectors (M = 1.941, STD = 0.102) and proximal effectors (M = 1.905, STD = 0.107). This result indicated that participants were able to achieve the goal pattern ratio predefined by the nature of the 1:2 coordination pattern between the effectors regardless of the proximity to the midline of the body (Fig 5).

#### Correlation

**Fig 6:**
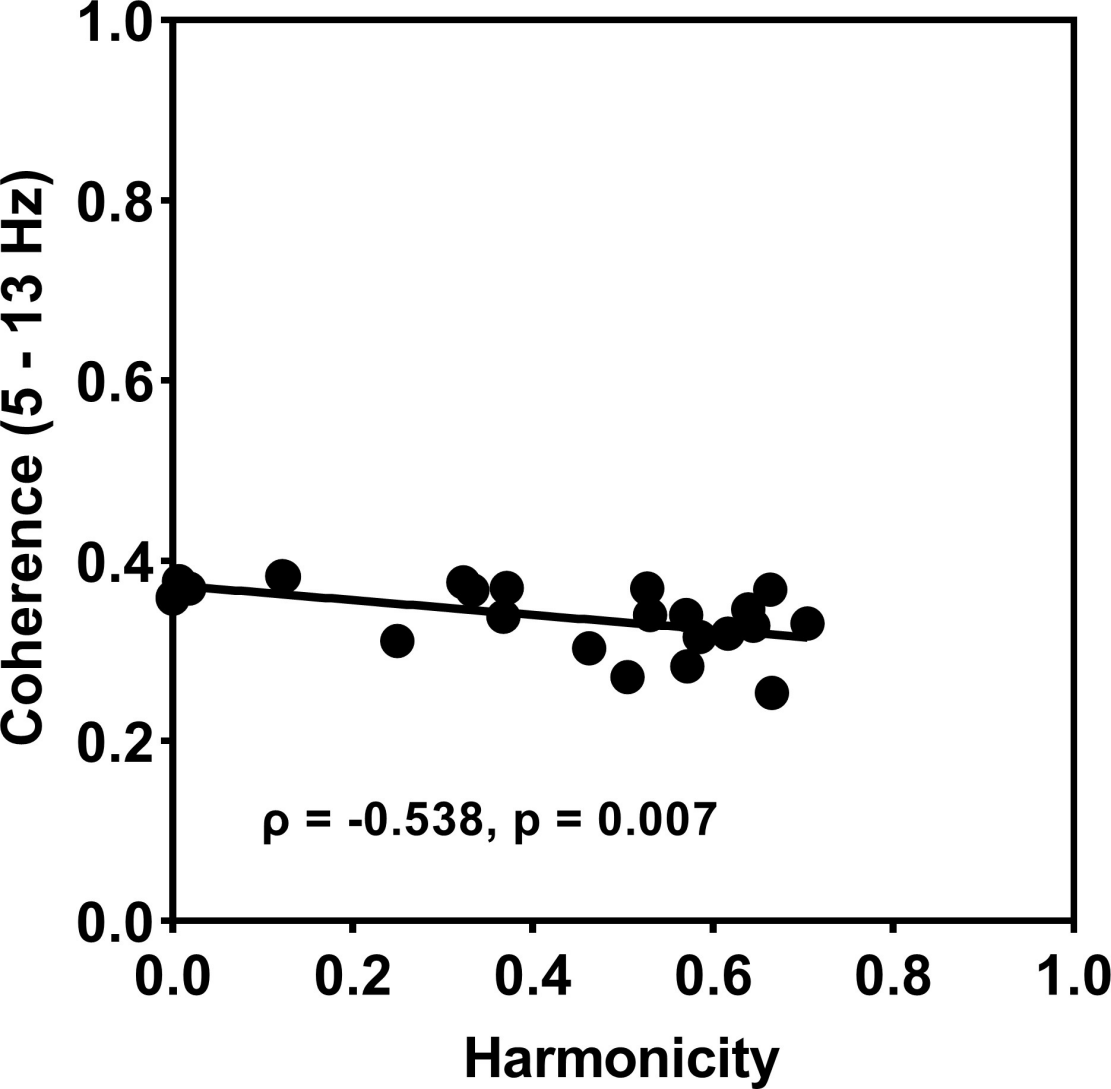
EMG-EMG coherence and harmonicity correlation. EMG-EMG coherence in the alpha bands is significantly correlated with harmonicity of non-dominant effector combined from both groups. The negative value of correlation coefficient indicates an inverse relationship between EMG-EMG coherence and harmonicity of non-dominant effector. The strength of the correlation is moderate to vigorous.

The Pearson correlation analysis indicated that harmonicity of the non-dominant effector is inversely correlated with EMG-EMG coherence (ρ = -0.548, p = 0.006 (2-tailed)) (Fig 6).

## Discussion

It has been hypothesized that the influence of neural crosstalk on bimanual coordination is stronger for proximal effectors than for distal effectors [13, 14]. The current investigation was designed to determine the effects of distal (FDI) and proximal (TBI) muscle activation on neural crosstalk when participants were required to produce a 1:2 multifrequency force pattern.

### Task performance

Research has indicated that a 1:2 multifrequency task can pose difficult challenges for the central nervous system (CNS) [16, 49, 75]. However, many of the difficulties associated with coordinating multifrequency patterns appear to be circumvented, or at least minimized, when integrated feedback information (e.g., Lissajous displays) is provided [76]. Based upon two global measures of goal attainment (i.e., inter-peak interval ratio and phase angle slope ratio), the results of the current investigation are consistent with previous research demonstrating the effectiveness of Lissajous displays in facilitating the performance of complex bimanual coordination patterns. Regardless of the effector group (distal, proximal) used to perform the task, participants could effectively produce the 1:2 multifrequency with Lissajous displays (Fig 5).

Despite the effective task performance with both distal and proximal effectors, the results indicated participants were more temporally accurate when performing the task with their distal effectors than with their proximal effectors. It is not surprising that individuals are more accurate with their distal (fingers) than proximal (arms) effectors, given that distal effectors (e.g., FDI) are often associated with the control of fine motor skills [77] while proximal effectors (e.g., TBI) are often associated with the control of gross motor skills [77].

In general, the accuracy of motor skills is often associated with how well the neural system can integrate sensory information with the desired motor outputs [78]. The process of perceptual-motor integration is often found to be greater for effectors involved in the control of fine motor skills than effectors controlling gross motor skills [78]. Previous research has also indicated that coordination of bimanual actions is predominantly dependent on the process of perceptual-motor integration [2, 7]. As such, distal effectors may have an advantage when required to produce precise actions when compared to proximal effectors. Indeed, research has indicated more accurate control of force with distal than proximal effectors [14, 79].

### Symmetric and asymmetric neural crosstalk

Although it is well documented that individuals are often more accurate when performing fine motor actions compared to gross motor actions [14, 79], the current investigation may provide behavioral and neurophysiological evidence to account for these differences. Due to neuroanatomical differences between the neural organization of distal and proximal effectors (e.g., a distinct connection between brain and body, number of commissural fibers connecting muscles through the CC and spinal cord, the influence of uncrossed ventromedial corticospinal tracts and crossed lateral corticospinal tracts) [37–42], proximal effectors are likely influenced more by neural crosstalk than distal effectors [13, 14]. Consistent with the possibility that proximal effectors are more susceptible to neural crosstalk, lower force harmonicity was observed for the non-dominant proximal effector than distal effector (Fig 4). Note, harmonicity is a measure that has been used to reflect neural crosstalk [4, 28]. The results indicated more adjustments, hesitation, and/or perturbations occurred in the non-dominant proximal effector than the non-dominant distal effector. However, harmonicity for the dominant effector was not significantly different between distal and proximal effectors (Fig 4). This result is consistent with the notion that neural crosstalk is asymmetric, with the dominant effector/hemisphere exerting a stronger influence on the non-dominant effector/hemisphere than vice versa [21, 23, 80, 81].

Numerous investigations have demonstrated performance asymmetries associated with limb dominance with the dominant limb more accurate and stable than the non-dominant limb [4, 21, 23, 33, 80–83]. It has been suggested that the dominant hemisphere exerts a stronger influence on the non-dominant limb than vice versa [21, 23, 80, 81]. Consistent with this view, researchers successfully simulated a neural crosstalk model for bimanual interference based on the left (non-dominant) effector receiving an attenuated mirror image of the commands dispatched to the right (dominant) effector [21]. The simulation successfully reproduced characteristics associated with asymmetric bimanual circle including a deterioration of the circular trajectories and a weakening of the phase coupling between the effectors when movement frequency was increased [21]. The results of the current investigation are consistent with the neural crosstalk model [21]. That is, distortions in the non-dominant limb force and force-velocity time series that were associated with the production of force by the dominant limb were observed. However, distortions in the forces produced by the dominant limb that could be attributed to forces produced by the non-dominant limb were not observed (Figs 3 and 4). It is important to note, however, that the current investigation used a multifrequency force task in which the dominant limb was assigned the faster frequency (1:2).

In an experiment designed to determine whether the influence of force produced by one limb on the contralateral limb was the result of the limb assigned the faster frequency or a bias associated with limb dominance, performance between a 1:2 and 2:1 multifrequency force task for right limb dominant participants was compared. The results indicated that the source of interference was not limited to limb assignment but also a function of limb dominance. A similar investigation used a 1:2 bimanual force task with symmetric and asymmetric force requirements [28]. The results indicated that distortions in the forces produced by the non-dominant effector that could be associated with forces produced by the dominant effector occurred regardless of the condition (symmetric, asymmetric) or which effector produced the greater force. However, distortions in the dominant effector were only observed when the non-dominant effector was required to produce 5 times more force than the dominant effector to perform the required coordination pattern. Overall, the results of this line of research indicate that neural crosstalk affects both effectors, however, it may manifest differently for the non-dominant and dominant limbs.

It is possible that individuals are more efficient at inhibiting, compensating, and/or overriding neural crosstalk with the dominant limb than with the non-dominant limb [82, 84, 85]. Support for such a possibility can be found in research indicating that bimanual interference can be reduced with extended practice [86, 87]. Note, however, the current investigation only provided participants approximately 5 minutes of practice with each effector group before the test trials. If, however, we consider the frequency in which the dominant limb is used in activities of daily living (i.e., amount of practice), it is possible that individuals have become more resistant to the effects of neural crosstalk with the dominant effector than with the non-dominant effector. Research should further explore this possibility.

### Muscle activation and neural crosstalk

The results of the EMG-EMG coherence analysis are also consistent with the possibility that proximal effectors are more susceptible to neural crosstalk than distal effectors. More specifically, EMG coherence between two homologous muscles was used as a measure of common neural input [11, 88]. Neural crosstalk is a mirror image command sent to homologous muscles of the contralateral effector [10, 21, 22]. It conveys the same information to both effectors from common neural input. Therefore, higher EMG coherence is consistent with higher levels of neural crosstalk. A recent experiment using EMG-EMG coherence indicated higher coherence between the EMG signals when two effectors were producing the same actions compared to when each effector produced disparate actions [11]. In the current investigation, the intermuscular EMG coherence was significantly higher for the proximal condition than the distal condition in the Alpha band (5–13 Hz) (Fig 5), supporting the notion that neural crosstalk is stronger for proximal effectors than distal effectors.

Significant EMG coherence in the Alpha band suggests a subcortical origin for neural crosstalk [11, 50]. Other studies have also highlighted the contribution of the subcortical structures (e.g., cerebellum, basal ganglia, and spinal interneurons) and uncrossed corticospinal tracts to the performance of bimanual tasks specified by coupling temporal features [13, 14, 39–42, 47, 48, 89–94]. The nature of the 1:2 bimanual coordination task requires the dominant effector to produce the actions twice as fast as the non-dominant effector. Thus, the successful performance of the 1:2 bimanual coordination task could be associated with how well the temporal relationship between the effectors is calibrated. Consequently, the subcortical areas may play a predominant role in calibrating the timing information during the current bimanual task [48].

To further understand the relationship between neural crosstalk and EMG coherence, a statistical analysis was performed to examine the correlation between the harmonicity in the non-dominant effector and the coherence values in the alpha band. The results indicated a significant inverse correlation between the two variables (Fig 6), suggesting that an increase in neural crosstalk is associated with greater force distortions in the non-dominant effector. This suggestion is consistent with several investigations demonstrating consistent and identifiable distortions in the non-dominant effector attributed to the activation and release of force by the dominant effector [4, 5, 26, 28, 35, 95].

**Fig 7:**
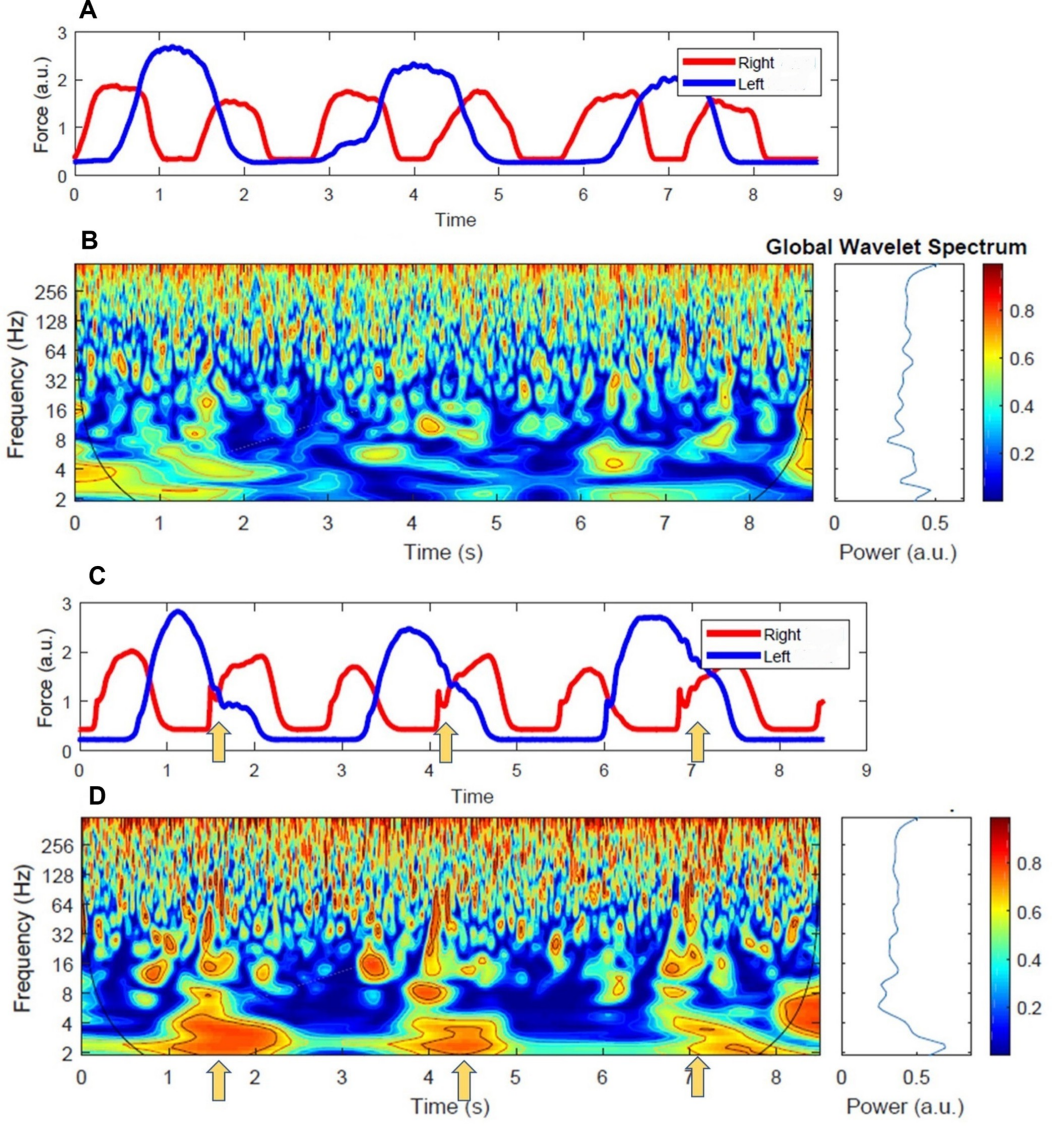
EMG analysis. (a) Sample non-dominant and dominant distal (FDI) effector EMG Amplitude, (b) the corresponding EMG-EMG Wavelet Spectrum for distal effector. (c) Sample non-dominant and dominant proximal (TBI) effector EMG Amplitude, (d) the corresponding EMG-EMG Wavelet Spectrum for proximal effector. Note, the arrows indicate areas where high coherence occurred relative to behavior and the color code represents the strength of the coherence on a scale of 0 (blue) to 1 (red).

The spectral-frequency graphs (Fig 7) show the dynamics of the coherence intensity in a trial. The color gradient indicates that the coherence was stronger for the proximal effectors than the distal effectors. Note, the color code represents the strength of the coherence on a scale of 0 (blue) to 1 (red). Furthermore, the periods associated with the most substantial coherences were intermittent rather than continuous. These periods appear to be coincident with the dominant effector’s activation and release of force (Fig 7; yellow arrows). From a theoretical standpoint, if some fraction of the force command from one effector is diverted to the other effector, then the same information would be conveyed to homologous muscles of both limbs when the command is dispatched [10, 21]. This would result in periods of shared neural drive with the initiation or release of the force command. This observation is consistent with the kinematic results of the current study (Fig 3) as well as previous work demonstrating force distortions in the force and force-velocity time series for the non-dominant effector that was associated with the activation and release of force by the dominant effector [4, 5, 11, 25]. In addition, research has indicated this influence continues to act on the non-dominant limb until the dominant limb achieves peak force velocity [4]. Interestingly, research examining the tri-phasic pattern of muscle activation during isometric contractions found that the initial burst in the agonist muscle terminates when peak velocity is achieved [96].

Although the current study did not indicate a cortical origin for neural crosstalk, cortical influence may play a role in mediating the synchronization of a bimanual task. A recent investigation using EEG-EMG coherence revealed a significant coherence in the Beta band during a discrete bimanual task [88]. Neural activity in the beta bands is consistent with a cortical influence. In addition, studies investigating bimanual performance among split-brain patients often demonstrate the importance of CC during bimanual actions [47, 48, 97, 98]; these studies also suggest the CC’s contribution is task-dependent. Thus, the properties associated with the task (easy vs. difficult; discrete vs. continuous) may produce different cortical and subcortical interactions when synchronizing bimanual actions. In contrast, careful observation of Fig 7, especially at the instants marked by the yellow arrows, shows greater pockets of significant coherence in the Beta band for proximal (Fig 7) than the distal effector (Fig 7). This observation suggests that the lack of statistical significance differences in the Beta bands between effector groups may have been caused by the intermittence of the frequency features of the signals. Future studies should attempt to consider time-dependent statistical analyses for group comparisons.

### Summary

The present experiment was designed to determine the effects of distal (FDI) and proximal (TBI) muscle activation on neural crosstalk. Participants were able to effectively produce the goal bimanual coordination pattern (1:2) with both proximal and distal effectors. However, spatiotemporal accuracy with greater when performed with the distal effectors (FDI) compared to proximal effectors (TBI). Consistent with the asymmetric nature of neural crosstalk, the analysis of harmonicity indicated greater distortion in force produced by the non-dominant effector for both effector groups. However, harmonicity was lower for the non-dominant effector in the proximal condition than for the distal effector indicating more bimanual interference for proximal than distal effectors. The intermuscular EMG coherence results showed significantly higher bimanual communication in the Alpha bands (5–13 Hz) for proximal than distal effectors. A correlation analysis between the intermuscular EMG coherence and harmonicity indicated that bimanual interference is inversely associated with bimanual communication. These results provide behavior and neurophysiological support that neural crosstalk is stronger for proximal than distal effectors and may have important implication for rehabilitation protocols designed for recovery of hand and arm function.

## References

[1] Kelso JA. Phase transitions and critical behavior in human bimanual coordination. Am J Physiol. 1984;246(6 Pt 2):R1000–4. doi: 10.1152/ajpregu.1984.246.6.R1000 6742155

[2] Buchanan JJ, Wang C. Overcoming the guidance effect in motor skill learning: feedback all the time can be beneficial. Exp Brain Res. 2012;219(2):305–20. doi: 10.1007/s00221-012-3092-x 22526952

[3] Fontaine RJ, Lee TD, Swinnen SP. Learning a new bimanual coordination pattern: reciprocal influences of intrinsic and to-be-learned patterns. Can J Exp Psychol. 1997;51(1):1–9. doi: 10.1037/1196-1961.51.1.1 9206321

[4] Kennedy DM, Boyle JB, Rhee J, Shea CH. Rhythmical bimanual force production: homologous and non-homologous muscles. Exp Brain Res. 2015;233(1):181–95. doi: 10.1007/s00221-014-4102-y 25248845

[5] Kennedy DM, Rhee J, Shea CH. Symmetrical and asymmetrical influences on force production in 1:2 and 2:1 bimanual force coordination tasks. Exp Brain Res. 2016;234(1):287–300. doi: 10.1007/s00221-015-4460-0 26466827

[6] Kovacs AJ, Buchanan JJ, Shea CH. Using scanning trials to assess intrinsic coordination dynamics. Neurosci Lett. 2009;455(3):162–7. doi: 10.1016/j.neulet.2009.02.046 19429113

[7] Kovacs AJ, Buchanan JJ, Shea CH. Bimanual 1:1 with 90 degrees continuous relative phase: difficult or easy! Exp Brain Res. 2009;193(1):129–36. doi: 10.1007/s00221-008-1676-2 19093104

[8] Kovacs AJ, Buchanan JJ, Shea CH. Impossible is nothing: 5:3 and 4:3 multi-frequency bimanual coordination. Exp Brain Res. 2010;201(2):249–59. doi: 10.1007/s00221-009-2031-y 19798488

[9] Kovacs AJ, Buchanan JJ, Shea CH. Perceptual and attentional influences on continuous 2:1 and 3:2 multi-frequency bimanual coordination. J Exp Psychol Hum Percept Perform. 2010;36(4):936–54. doi: 10.1037/a0019259 20695710

[10] Swinnen SP. Intermanual coordination: from behavioural principles to neural-network interactions. Nat Rev Neurosci. 2002;3(5):348–59. doi: 10.1038/nrn807 11988774

[11] Wang Y, Neto OP, Davis MM, Kennedy DM. The effect of inherent and incidental constraints on bimanual and social coordination. Exp Brain Res. 2021;239(7):2089–105. doi: 10.1007/s00221-021-06114-8 33929601

[12] Zanone PG, Kelso JA. Evolution of behavioral attractors with learning: nonequilibrium phase transitions. J Exp Psychol Hum Percept Perform. 1992;18(2):403–21. doi: 10.1037//0096-1523.18.2.403 1593227

[13] Aune MA, Loras H, Djuvsland A, Ingvaldsen RP, Aune TK. More Pronounced Bimanual Interference in Proximal Compared to Distal Effectors of the Upper Extremities. Front Psychol. 2020;11:544990. doi: 10.3389/fpsyg.2020.544990 33192790 PMC7652815

[14] Aune MA, Loras H, Nynes A, Aune TK. Bilateral Interference in Motor Performance in Homologous vs. Non-homologous Proximal and Distal Effectors. Front Psychol. 2021;12:680268. doi: 10.3389/fpsyg.2021.680268 34322064 PMC8310955

[15] Lee TD, Swinnen SP, Verschueren S. Relative Phase Alterations During Bimanual Skill Acquisition. J Mot Behav. 1995;27(3):263–74. doi: 10.1080/00222895.1995.9941716 12529237

[16] Swinnen SP, Dounskaia N, Walter CB, Serrien DJ. Preferred and induced coordination modes during the acquisition of bimanual movements with a 2: 1 frequency ratio. J Exp Psychol Hum Percept Perform. 1997;23(4):1087.

[17] Swinnen SP, Van Langendonk L, Verschueren S, Peeters G, Dom R, De Weerdt W. Interlimb coordination deficits in patients with Parkinson’s disease during the production of two-joint oscillations in the sagittal plane. Mov Disord. 1997;12(6):958–68. doi: 10.1002/mds.870120619 9399221

[18] Swinnen SP, Wenderoth N. Two hands, one brain: cognitive neuroscience of bimanual skill. Trends Cogn Sci. 2004;8(1):18–25. doi: 10.1016/j.tics.2003.10.017 14697399

[19] Carson RG. Neural pathways mediating bilateral interactions between the upper limbs. Brain Res Brain Res Rev. 2005;49(3):641–62. doi: 10.1016/j.brainresrev.2005.03.005 15904971

[20] Swinnen SP, Gooijers J. Bimanual coordination. Brain mapping: an encyclopedic reference. 2015;2:475–82.

[21] Cattaert D, Semjen A, Summers JJ. Simulating a neural cross-talk model for between-hand interference during bimanual circle drawing. Biol Cybern. 1999;81(4):343–58. doi: 10.1007/s004220050567 10541937

[22] Cardoso de Oliveira S. The neuronal basis of bimanual coordination: recent neurophysiological evidence and functional models. Acta Psychol (Amst). 2002;110(2–3):139–59. doi: 10.1016/s0001-6918(02)00031-8 12102103

[23] Kagerer FA, Summers JJ, Semjen A. Instabilities during antiphase bimanual movements: are ipsilateral pathways involved? Exp Brain Res. 2003;151(4):489–500. doi: 10.1007/s00221-003-1496-3 12845510

[24] Marteniuk R, MacKenzie C, Baba D. Bimanual movement control: Information processing and interaction effects. The Quarterly Journal of Experimental Psychology Section A. 1984;36(2):335–65.

[25] Diaz-Artiles A, Wang Y, Davis MM, Abbott R, Keller N, Kennedy DM. The Influence of Altered-Gravity on Bimanual Coordination: Retention and Transfer. Front Physiol. 2022:2378. doi: 10.3389/fphys.2021.794705 35069255 PMC8777123

[26] Kennedy DM, Boyle JB, Wang C, Shea CH. Bimanual force control: cooperation and interference? Psychol Res. 2016;80(1):34–54. doi: 10.1007/s00426-014-0637-6 25481636

[27] Kennedy DM, Wang C, Panzer S, Shea CH. Continuous scanning trials:Transitioning through the attractor landscape. Neurosci Lett. 2016;610:66–72. doi: 10.1016/j.neulet.2015.10.073 26546133

[28] Kennedy DM, Rhee J, Jimenez J, Shea CH. The influence of asymmetric force requirements on a multi-frequency bimanual coordination task. Hum Mov Sci. 2017;51:125–37. doi: 10.1016/j.humov.2016.12.007 28027462

[29] Treffner PJ, Turvey M. Handedness and the asymmetric dynamics of bimanual rhythmic coordination. J Exp Psychol Hum Percept Perform. 1995;21(2):318.

[30] Peters M. Attentional asymmetries during concurrent bimanual performance. The Quarterly Journal of Experimental Psychology. 1981;33(1):95–103.

[31] Carson RG, Thomas J, Summers JJ, Walters MR, Semjen A. The dynamics of bimanual circle drawing. The Quarterly Journal of Experimental Psychology Section A. 1997;50(3):664–83. doi: 10.1080/713755721 9314729

[32] Gooijers J, Caeyenberghs K, Sisti HM, Geurts M, Heitger MH, Leemans A, et al. Diffusion tensor imaging metrics of the corpus callosum in relation to bimanual coordination: effect of task complexity and sensory feedback. Hum Brain Mapp. 2013;34(1):241–52. doi: 10.1002/hbm.21429 22021056 PMC6869984

[33] Semjen A, Summers JJ, Cattaert D. Hand coordination in bimanual circle drawing. J Exp Psychol Hum Percept Perform. 1995;21(5):1139.

[34] Byblow WD, Chua R, Bysouth-Young DF, Summers JJ. Stabilisation of bimanual coordination through visual coupling. Human Movement Science. 1999;18(2–3):281–305.

[35] Panzer S, Kennedy D, Leinen P, Pfeifer C, Shea C. Bimanual coordination associated with left-and right-hand dominance: testing the limb assignment and limb dominance hypothesis. Exp Brain Res. 2021;239(5):1595–605. doi: 10.1007/s00221-021-06082-z 33748885 PMC8144160

[36] Swinnen SP, Jardin K, Meulenbroek R. Between-limb asynchronies during bimanual coordination: effects of manual dominance and attentional cueing. Neuropsychologia. 1996;34(12):1203–13. doi: 10.1016/0028-3932(96)00047-4 8951832

[37] Pandya DN, Vignolo LA. Intra- and interhemispheric projections of the precentral, premotor and arcuate areas in the rhesus monkey. Brain Res. 1971;26(2):217–33. 4993847

[38] Jenny AB. Commissural projections of the cortical hand motor area in monkeys. J Comp Neurol. 1979;188(1):137–45. doi: 10.1002/cne.901880111 115906

[39] Kuypers HG. The motor system and the capacity to execute highly fractionated distal extremity movements. Electroencephalogr Clin Neurophysiol Suppl. 1978(34):429–31. 108079

[40] Palmer E, Ashby P. Corticospinal projections to upper limb motoneurones in humans. J Physiol. 1992;448:397–412. doi: 10.1113/jphysiol.1992.sp019048 1593472 PMC1176206

[41] Lemon RN. An enduring map of the motor cortex. Exp Physiol. 2008;93(7):798–802. doi: 10.1113/expphysiol.2007.039081 18562475

[42] Lemon RN, Kirkwood PA, Maier MA, Nakajima K, Nathan P. Direct and indirect pathways for corticospinal control of upper limb motoneurons in the primate. Prog Brain Res. 2004;143:263–79. doi: 10.1016/S0079-6123(03)43026-4 14653171

[43] Rouiller EM, Babalian A, Kazennikov O, Moret V, Yu XH, Wiesendanger M. Transcallosal connections of the distal forelimb representations of the primary and supplementary motor cortical areas in macaque monkeys. Exp Brain Res. 1994;102(2):227–43. doi: 10.1007/BF00227511 7705502

[44] Jankowska E, Edgley SA, Krutki P, Hammar I. Functional differentiation and organization of feline midlumbar commissural interneurones. J Physiol. 2005;565(Pt 2):645–58. doi: 10.1113/jphysiol.2005.083014 15817636 PMC1464510

[45] Pierrot-Deseilligny E, Burke D. The circuitry of the human spinal cord: its role in motor control and movement disorders: Cambridge university press; 2005.

[46] Seidler RD. Neural correlates of motor learning, transfer of learning, and learning to learn. Exerc Sport Sci Rev. 2010;38(1):3–9. doi: 10.1097/JES.0b013e3181c5cce7 20016293 PMC2796204

[47] Franz EA, Eliassen JC, Ivry RB, Gazzaniga MS. Dissociation of spatial and temporal coupling in the bimanual movements of callosotomy patients. Psychol Sci. 1996;7(5):306–10.

[48] Tuller B, Kelso JA. Environmentally-specified patterns of movement coordination in normal and split-brain subjects. Exp Brain Res. 1989;75(2):306–16. doi: 10.1007/BF00247936 2721610

[49] Puttemans V, Wenderoth N, Swinnen SP. Changes in brain activation during the acquisition of a multifrequency bimanual coordination task: from the cognitive stage to advanced levels of automaticity. J Neurosci. 2005;25(17):4270–8. doi: 10.1523/JNEUROSCI.3866-04.2005 15858053 PMC6725124

[50] Boonstra TW, van Wijk BC, Praamstra P, Daffertshofer A. Corticomuscular and bilateral EMG coherence reflect distinct aspects of neural synchronization. Neurosci Lett. 2009;463(1):17–21. doi: 10.1016/j.neulet.2009.07.043 19619608

[51] Heuer H, Kleinsorge T, Spijkers W, Steglich W. Static and phasic cross-talk effects in discrete bimanual reversal movements. J Mot Behav. 2001;33(1):67–85. doi: 10.1080/00222890109601904 11265059

[52] Dideriksen JL, Negro F, Falla D, Kristensen SR, Mrachacz-Kersting N, Farina D. Coherence of the Surface EMG and Common Synaptic Input to Motor Neurons. Front Hum Neurosci. 2018;12:207. doi: 10.3389/fnhum.2018.00207 29942254 PMC6004394

[53] Pereira R, Freire IV, Cavalcanti CV, Luz CP, Neto OP. Hand dominance during constant force isometric contractions: evidence of different cortical drive commands. Eur J Appl Physiol. 2012;112(8):2999–3006. doi: 10.1007/s00421-011-2278-4 22170017

[54] Brown P. Cortical drives to human muscle: the Piper and related rhythms. Prog Neurobiol. 2000;60(1):97–108. doi: 10.1016/s0301-0082(99)00029-5 10622378

[55] Grosse P, Brown P. Acoustic startle evokes bilaterally synchronous oscillatory EMG activity in the healthy human. J Neurophysiol. 2003;90(3):1654–61. doi: 10.1152/jn.00125.2003 12750424

[56] Pollock A, St George B, Fenton M, Firkins L. Top ten research priorities relating to life after stroke. Lancet Neurol. 2012;11(3):209. doi: 10.1016/S1474-4422(12)70029-7 22341029

[57] Lodha N, Patten C, Coombes SA, Cauraugh JH. Bimanual force control strategies in chronic stroke: finger extension versus power grip. Neuropsychologia. 2012;50(11):2536–45. doi: 10.1016/j.neuropsychologia.2012.06.025 22781814

[58] Rose DK, Winstein CJ. Bimanual training after stroke: are two hands better than one? Top Stroke Rehabil. 2004;11(4):20–30. doi: 10.1310/NCB1-JWAA-09QE-7TXB 15592987

[59] Stewart KC, Cauraugh JH, Summers JJ. Bilateral movement training and stroke rehabilitation: a systematic review and meta-analysis. J Neurol Sci. 2006;244(1–2):89–95. doi: 10.1016/j.jns.2006.01.005 16476449

[60] Hallett M. Plasticity of the human motor cortex and recovery from stroke. Brain Res Rev. 2001;36(2–3):169–74. doi: 10.1016/s0165-0173(01)00092-3 11690613

[61] Harmsen WJ, Bussmann JB, Selles RW, Hurkmans HL, Ribbers GM. A Mirror Therapy-Based Action Observation Protocol to Improve Motor Learning After Stroke. Neurorehabil Neural Repair. 2015;29(6):509–16. doi: 10.1177/1545968314558598 25416737

[62] Qian Q, Nam C, Guo Z, Huang Y, Hu X, Ng SC, et al. Distal versus proximal-an investigation on different supportive strategies by robots for upper limb rehabilitation after stroke: a randomized controlled trial. J Neuroeng Rehabil. 2019;16(1):1–16. 31159822 10.1186/s12984-019-0537-5 PMC6545723

[63] Turton A, Wroe S, Trepte N, Fraser C, Lemon RN. Contralateral and ipsilateral EMG responses to transcranial magnetic stimulation during recovery of arm and hand function after stroke. Electroencephalogr Clin Neurophysiol. 1996;101(4):316–28. doi: 10.1016/0924-980x(96)95560-5 8761041

[64] Hesse S, Mehrholz J, Werner C. Robot-assisted upper and lower limb rehabilitation after stroke: walking and arm/hand function. Dtsch Arztebl Int. 2008;105(18):330–6. doi: 10.3238/arztebl.2008.0330 19629252 PMC2707632

[65] Volpe BT, Ferraro M, Lynch D, Christos P, Krol J, Trudell C, et al. Robotics and other devices in the treatment of patients recovering from stroke. Curr Atheroscler Rep. 2004;6(4):314–9. doi: 10.1007/s11883-004-0064-z 15191707

[66] Oldfield RC. The assessment and analysis of handedness: the Edinburgh inventory. Neuropsychologia. 1971;9(1):97–113. doi: 10.1016/0028-3932(71)90067-4 5146491

[67] Kovacs AJ, Wang Y, Kennedy DM. Accessing interpersonal and intrapersonal coordination dynamics. Exp Brain Res. 2020;238(1):17–27. doi: 10.1007/s00221-019-05676-y 31754737

[68] Guiard Y. On Fitts’s and Hooke’s laws: Simple harmonic movement in upper-limb cyclical aiming. Acta Psychol (Amst). 1993;82(1–3):139–59. doi: 10.1016/0001-6918(93)90009-g 8475763

[69] Laine CM, Valero-Cuevas FJ. Intermuscular coherence reflects functional coordination. J Neurophysiol. 2017;118(3):1775–83. doi: 10.1152/jn.00204.2017 28659460 PMC5596118

[70] Baweja HS, Patel BK, Neto OP, Christou EA. The interaction of respiration and visual feedback on the control of force and neural activation of the agonist muscle. Hum Mov Sci. 2011;30(6):1022–38. doi: 10.1016/j.humov.2010.09.007 21546109 PMC3202062

[71] Marzullo TC, Lehmkuhle MJ, Gage GJ, Kipke DR. Development of closed-loop neural interface technology in a rat model: combining motor cortex operant conditioning with visual cortex microstimulation. IEEE Trans Neural Syst Rehabil Eng. 2010;18(2):117–26. doi: 10.1109/TNSRE.2010.2041363 20144922 PMC2941890

[72] Torrence C, Compo GP. A practical guide to wavelet analysis. Bulletin of the American Meteorological society. 1998;79(1):61–78.

[73] Neto OP, Pinto IR, Pinto O Jr. The relationship between thunderstorm and solar activity for Brazil from 1951 to 2009. Journal of Atmospheric and Solar-Terrestrial Physics. 2013;98:12–21.

[74] Grinsted A, Moore JC, Jevrejeva S. Application of the cross wavelet transform and wavelet coherence to geophysical time series. Nonlinear processes in geophysics. 2004;11(5/6):561–6.

[75] Summers JJ, Davis AS, Byblow WD. The acquisition of bimanual coordination is mediated by anisotropic coupling between the hands. Human movement science. 2002;21(5–6):699–721. doi: 10.1016/s0167-9457(02)00151-3 12620717

[76] Shea CH, Buchanan JJ, Kennedy DM. Perception and action influences on discrete and reciprocal bimanual coordination. Psychon Bull Rev. 2016;23(2):361–86. doi: 10.3758/s13423-015-0915-3 26282829

[77] Piek JP, Dawson L, Smith LM, Gasson N. The role of early fine and gross motor development on later motor and cognitive ability. Hum Mov Sci. 2008;27(5):668–81. doi: 10.1016/j.humov.2007.11.002 18242747

[78] Jenkinson J, Hyde T, Ahmad S. Building blocks for learning occupational therapy approaches: Practical strategies for the inclusion of special needs in primary school: John Wiley & Sons; 2008.

[79] Brychta P, Hojk V, Hrubý J, Pilc J. Influence of fine motor skill on accuracy of measurements using a handheld sliding caliper at adolescents group aged 19–20. Technological Engineering. 2017;14(1):20–3.

[80] Aramaki Y, Honda M, Okada T, Sadato N. Neural correlates of the spontaneous phase transition during bimanual coordination. Cereb Cortex. 2006;16(9):1338–48. doi: 10.1093/cercor/bhj075 16306323

[81] Maki Y, Wong KFK, Sugiura M, Ozaki T, Sadato N. Asymmetric control mechanisms of bimanual coordination: an application of directed connectivity analysis to kinematic and functional MRI data. Neuroimage. 2008;42(4):1295–304. doi: 10.1016/j.neuroimage.2008.06.045 18674627

[82] de Poel HJ, Peper CLE, Beek PJ. Handedness-related asymmetry in coupling strength in bimanual coordination: furthering theory and evidence. Acta Psychol (Amst). 2007;124(2):209–37. doi: 10.1016/j.actpsy.2006.03.003 16777042

[83] Peters M. Performance of a rubato-like task: When two things cannot be done at the same time. Music Perception. 1985;2(4):471–82.

[84] Serrien DJ, Cassidy MJ, Brown P. The importance of the dominant hemisphere in the organization of bimanual movements. Hum Brain Mapp. 2003;18(4):296–305. doi: 10.1002/hbm.10086 12632467 PMC6871910

[85] Stinear JW, Byblow WD. Rhythmic bilateral movement training modulates corticomotor excitability and enhances upper limb motricity poststroke: a pilot study. J Clin Neurophysiol. 2004;21(2):124–31. doi: 10.1097/00004691-200403000-00008 15284604

[86] Albert NB, Ivry RB. The persistence of spatial interference after extended training in a bimanual drawing task. Cortex. 2009;45(3):377–85. doi: 10.1016/j.cortex.2007.11.012 18718578 PMC2677900

[87] Sosnik R. Practice makes bimanual interference imperfect–On the way to the generation of bimanual motion primitives. Cortex. 2010;46(2):264–7. doi: 10.1016/j.cortex.2009.02.008 19321164

[88] Chen YT, Li S, Magat E, Zhou P, Li S. Motor Overflow and Spasticity in Chronic Stroke Share a Common Pathophysiological Process: Analysis of Within-Limb and Between-Limb EMG-EMG Coherence. Front Neurol. 2018;9:795. doi: 10.3389/fneur.2018.00795 30356703 PMC6189334

[89] Graybiel AM. Building action repertoires: memory and learning functions of the basal ganglia. Curr Opin Neurobiol. 1995;5(6):733–41. doi: 10.1016/0959-4388(95)80100-6 8805417

[90] Ivry RB, Keele SW. Timing functions of the cerebellum. J Cogn Neurosci. 1989;1(2):136–52. doi: 10.1162/jocn.1989.1.2.136 23968462

[91] Grillner S. Neurobiological bases of rhythmic motor acts in vertebrates. Science. 1985;228(4696):143–9. doi: 10.1126/science.3975635 3975635

[92] Franz EA. Bimanual action representation: A window to human evolution. Taking action: Cognitive neuroscience perspectives on intentional acts. 2003:259–88.

[93] Donchin O, de Oliveira SC, Vaadia E. Who tells one hand what the other is doing: the neurophysiology of bimanual movements. Neuron. 1999;23(1):15–8. doi: 10.1016/s0896-6273(00)80748-5 10402189

[94] Gazzaniga MS, Sperry RW. Language after section of the cerebral commissures. Brain. 1967;90(1):131–48. doi: 10.1093/brain/90.1.131 6023071

[95] Leinen P, Vieluf S, Kennedy D, Aschersleben G, Shea CH, Panzer S. Life span changes: Performing a continuous 1:2 bimanual coordination task. Hum Mov Sci. 2016;46:209–20. doi: 10.1016/j.humov.2016.01.004 26800250

[96] Gordon J, Ghez C. EMG patterns in antagonist muscles during isometric contraction in man: relations to response dynamics. Exp Brain Res. 1984;55(1):167–71. doi: 10.1007/BF00240511 6745347

[97] Serrien DJ, Brown P. The functional role of interhemispheric synchronization in the control of bimanual timing tasks. Exp Brain Res. 2002;147(2):268–72. doi: 10.1007/s00221-002-1253-z 12410342

[98] Serrien DJ, Ivry RB, Swinnen SP. Dynamics of hemispheric specialization and integration in the context of motor control. Nat Rev Neurosci. 2006;7(2):160–6. doi: 10.1038/nrn1849 16429125

